# Lower thoracic syndrome

**DOI:** 10.12669/pjms.333.12340

**Published:** 2017

**Authors:** Muhammad Nazim Farooq

**Affiliations:** Dr. Muhammad Nazim Farooq, Islamabad College of Physiotherapy, Margalla Institute of Health Sciences, Rawalpindi, Pakistan

**Keywords:** Mobilization, Pain, Paresthesia, Sympathetic nervous system, Thoracic spine

## Abstract

The role of thoracic spine related dysfunction in producing lower extremity symptoms is not clear. This case study describes the assessment and treatment of a patient with low back pain and bilateral lower extremity (BLE) symptoms. It was found that patient education about postural awareness and passive mobilization are valuable aids to decrease BLE symptoms due to sympathetic nervous system (SNS) dysfunction and lower thoracic hypomobility. The clinicians need to consider examination and treatment of the lower thoracic area in patients with BLE symptoms. More research is required to explore the role of SNS dysfunction in producing BLE symptoms.

## INTRODUCTION

Patients having bilateral lower extremity (BLE) symptoms are usually seen by physiotherapists. These symptoms may include pain, pins and needles, tiredness, heaviness feelings, lower limb coldness and signs of neurological deficits. Clinicians often missed or misdiagnosed the symptoms arising from lower thoracic region as lumbar in nature and the patients’ do not recover or partially recover. The role of the sympathetic nervous system (SNS) for producing upper extremity symptoms has already been established as “the T4 syndrome”.^1^,^2^ However, the possible pathophysiological mechanisms producing BLE symptoms due to the involvement of thoracic spine are not clear.^3^ Many studies have already established the effectiveness of manual therapy in restoring the sympathetic functions.^3^,^4^

In this case report a 45 years old man has been discussed who presented with BLE symptoms giving suspicion of lower thoracic SNS dysfunction. The patient assessment, treatment and clinical interpretation of the symptoms have been discussed in this report.

### Subjective examination

Mr. A was referred to Physiotherapy center by an orthopedic surgeon, with complaint of low back pain (LBP) and BLE symptoms consisting of generalized pain and heaviness feelings which were usually preceded by tingling in feet. The symptoms were worse in left leg and were intermittent. His both legs become stiff in the morning and felt tired and heavy in the afternoon and at the end of the day.

Activities that aggravates his symptoms include bending forward, sitting for more than half an hour, sleeping, getting up from sitting position. Walking and shaking legs eases his symptoms once exacerbated. Sometimes massage with oil also relieved his symptoms.

Symptoms started about five months earlier as LBP without any clear cause. Later, he felt heavy and tired feeling in his left leg. Pain and paresthesia started in left leg three months after LBP. About one month ago he felt the same symptoms in his right leg as well. He was a computer operator and relate his symptoms with the second job which he started seven months ago. Symptoms got worse over time and his sleep was affected as well.

He went to an orthopedic consultant who performed an X-ray and MRI after examining him. X-ray showed minor degenerative changes at L3-4 and L4-5 levels. MRI report was normal. He had treatment from that consultant for three weeks but did not improve significantly.

The patient general health was good and he had no weight loss in the last few months. He had no complaint of bladder and bowl functions. His symptoms were not provoked by coughing and sneezing.

### Physical examination

On observation Mr. A was found to have good standing posture but with a reduced lumbar lordosis. When he was asked to sit, he adopted a slumped position. On palpation left leg was found cold. The range of right side flexion and right rotation was normal and painless. Left rotation, left side flexion and extension were found limited but without pain. Flexion was slightly limited and also without pain. Left rotation in the full flexed position was limited and painful. Quadrant test on left side produced pain in the lower lumbar region. But it was different from the patient’s actual LBP. Passive physiological intervertebral movements (PPIVMs) found decrease left rotation at T10-11 and T11-12. Unilateral postero-anterior (UPA) accessory movement revealed tenderness at T10 and T11 and loss of range of movement (ROM) at T10, T11 and T12. Straight leg raise test was normal on both sides. No deficit was found on testing the patient’s sensation, muscle strength and reflexes. However, the sympathetic slump test (SST) provoked the LBP and revealed limited left rotation. Sacroiliac and hip joints were found normal on performing screening tests.

### Clinical interpretation

The patient’s description of his pain, paresthesia and heaviness in non-dermatomal pattern may indicate a thoracic dysfunction.^2^ Night pain and paresthesia is also considered common in SNS dysfunctions.^1^ The relationship between SNS and thoracic spine has already been established in literature.^5^ UPA pressure produced tenderness at T10 and T11. Provocation of pain on palpation has been found as a reliable way to identify structures at fault and a key factor in clinical decision making.^6^ Furthermore, SST reproduced the LBP. The primary hypothesis of SNS dysfunction along with hypomobility of thoracic segments T10-12 was made based on available information.

The possibility of space occupying lesions and other serious pathology was ruled out because all questions about red flags were negative and the MRI scans of the patient were normal. The inflammatory condition was not expected because there was no increase pain on waking up in morning. Signs suggesting lumbar disc pathology were not found.

### Treatment

Total five treatment sessions were provided over a period of eighteen days. Subjective and objective findings were used to monitor the patient’s progress.

### Session I

Left rotation PPIVMs were applied at lower thoracic segments in side lying position. On reassessment, the range of left rotation and flexion-left rotation was improved slightly. But there was no improvement in pain and leg symptoms.

### Session II-IV

First, two sets of grade-III central postero-anterior (CPA) oscillatory movement was applied in prone lying with each set of two minutes duration. On reassessment slight improvement in flexion and left rotation range was observed only. There was no change in LBP and leg symptoms. Then two sets of grade-III UPA movement was applied in prone lying at T10-12 with each set of two minutes duration. Reassessment revealed improvement in LBP and the ranges of all active and passive movements were improved. The same technique was applied again on 3^rd^ and 4^th^ sessions. The patient was also educated about postural correction and for avoiding sustained positions of bending with rotation. In addition, the patient was provided with home exercises program including ten repetitions of left thoracic rotation and lumbar extension exercises three times a day.

### Session V

The patient was free of all symptoms except mild tenderness after sitting for long time. No more mobilization movements were provided in this treatment session. The patient was advised to take special care of his posture and perform home exercises to maintain mobility.

## DISCUSSION

In this case study, LBP and BLE symptoms of a patient were alleviated by mobilization of lower thoracic spine. The findings of this study are consistent with other studies.^4^,^5^ The thoracic spine role in producing neck and upper limb symptoms has already been investigated.^1^,^2^ Mobilization techniques have been found effective to relieve symptoms in such cases.^3^,^4^ Legs have sympathetic supply from T10-L2 levels. Therefore, SNS and lower thoracic dysfunctions can produce symptoms in legs and lumbar area.

Passive mobilization may stimulate the SNS and activate the descending pain inhibitory mechanism which increases sympathetic activity and decreases pain due to the release of noradrenaline from the dorsal periaqueductal grey area.^3^ The relief of symptoms may also be the result of direct stimulation of the local sympathetic fibers by the mobilization.^7^ Furthermore, a close relationship has been found between sympathetic excitation and hypoalgesia,^8^ which may support the use of mobilization for the treatment of thoracic syndrome.

In addition to passive mobilization, patient education about postural correction and home exercises plan were also used to maintain mobility and unload the sympathetic trunk to reduce leg and back symptoms. Many studies have found the combination of patient education and mobilization a valuable aid towards dealing patients with long-lasting symptoms.^9^ It has been stated that sympathetic trunk commonly get loaded by adopting flexed and rotated postures^10^ and sustaining these extreme positions for prolong periods of time may produce ischemic changes in sympathetic chain.^1^ Pain and paresthesia may occur in the extremities due to irritation of the SNS. Therefore, avoiding the extreme positions for prolong periods may reduce loading and ischemia of sympathetic chain and stimulate its activity. As a result the symptoms produced by sympathetic dysfunction get relieved.

## CONCLUSION

This case study found that passive mobilization combined with patient education to unload the sympathetic trunk are useful to decrease the BLE symptoms in patients having SNS dysfunction with associated lower thoracic hypomobility. The examination and treatment of the lower thoracic spine needs to be considered by the clinicians in patients having BLE symptoms as the thoracic dysfunction might be the causative factor. Further research exploring the role of SNS dysfunction in producing BLE symptoms needs to be done.
